# Epidemic and Nonepidemic Multidrug-Resistant *Enterococcus faecium*

**DOI:** 10.3201/eid0909.020383

**Published:** 2003-09

**Authors:** Helen L. Leavis, Rob J.L. Willems, Janetta Top, Emile Spalburg, Ellen M. Mascini, Ad C. Fluit, Andy Hoepelman, Albert J. de Neeling, Marc J.M. Bonten

**Affiliations:** * University Medical Center Utrecht, Utrecht, the Netherlands; † National Institute of Public Health and the Environment (RIVM), Bilthoven, the Netherlands

**Keywords:** *Enterococcus faecium*, antibiotic resistance, vancomycin, vancomycin-resistance, *esp* gene, ampicillin resistance, epidemiology

## Abstract

The epidemiology of vancomycin-resistant *Enterococcus faecium* (VREF) in Europe is characterized by a large community reservoir. In contrast, nosocomial outbreaks and infections (without a community reservoir) characterize VREF in the United States. Previous studies demonstrated host-specific genogroups and a distinct genetic lineage of VREF associated with hospital outbreaks, characterized by the variant *esp*-gene and a specific allele-type of the *purK* housekeeping gene (*purK*1). We investigated the genetic relatedness of *vanA* VREF (n=108) and vancomycin-susceptible *E. faecium* (VSEF) (n=92) from different epidemiologic sources by genotyping, susceptibility testing for ampicillin, sequencing of *purK*1, and testing for presence of *esp*. Clusters of VSEF fit well into previously described VREF genogroups, and strong associations were found between VSEF and VREF isolates with resistance to ampicillin, presence of *esp*, and *purK*1. Genotypes characterized by presence of *esp*, *purK*1, and ampicillin resistance were most frequent among outbreak-associated isolates and almost absent among community surveillance isolates. Vancomycin-resistance was not specifically linked to genogroups. VREF and VSEF from different epidemiologic sources are genetically related; evidence exists for nosocomial selection of a subtype of *E. faecium*, which has acquired vancomycin-resistance through horizontal transfer.

*Enterococcus faecium* has become an important nosocomial pathogen, especially in immunocompromised patients, creating serious limitations in treatment options because of cumulative resistance to antimicrobial agents (*1*). In the United States, the emergence of nosocomial *E. faecium* infections was characterized by increasing resistance to ampicillin in the 1980s and a rapid increase of vancomycin resistance in the next decade (*1*,*2*). The emergence of vancomycin-resistant *E. faecium* (VREF) in the United States illustrates the transmission capacities of bacteria and the possibility of a postantibiotic era for nosocomial infections in critically ill patients.

The global epidemiology of VREF is not well understood. In the United States, prevalences of colonization and infection are high among hospitalized patients, but a community reservoir of VREF in healthy persons or animals seems to be absent (*3*,*4*). In contrast, in Europe, colonization and infection rates within hospitals remain low, although colonization among healthy persons and animals is prevalent (*5*–*10*).

Previous studies suggested host-specificity of VREF genogroups (*11*), and isolates associated with nosocomial outbreaks seemed to be genetically distinct from nonepidemic VREF isolated from humans and animals (*12*). The differences between epidemic and nonepidemic isolates were based on genetic relatedness, as determined by amplified fragment length polymorphism analysis (AFLP), and the presence of an identical sequence of the *purK* housekeeping gene in epidemic strains (*12*). A recently developed multilocus sequence typing scheme for *E. faecium* confirmed that epidemic isolates belonged to a specific genetic lineage (*13*). Moreover, a variant of the *esp* gene, which has been found to be more prevalent among isolates of *E. faecalis* associated with infections (*14*), was found in all but one epidemic hospital-derived VREF isolate and not among community-derived VREF (*12*). Subsequently, other investigators described the variant *esp* gene in vancomycin-susceptible *E. faecium* (VSEF), and this gene appeared to be predominantly present among clinical isolates (*15*–*18*).These findings suggest the existence of a specific subpopulation of *E. faecium*, comprising both VREF as well as VSEF, associated with hospital outbreaks and infections.

In this study, we further investigated the genetic relationship between VREF and VSEF isolates, derived from different epidemiologic sources, such as hospital outbreaks, infections, and colonization among hospitalized patients and healthy persons. The genetic relatedness was linked to the presence of the variant *esp* gene and antibiotic resistance to ampicillin and vancomycin. On the basis of our findings, we constructed an evolutionary scheme describing the sequential steps in the development and selection of ampicillin- and vancomycin-resistant *E. faecium* strains.

## Materials and Methods

### Bacterial Strains and Growth Conditions

Isolates of VREF (n=108) were collected from nosocomial epidemics (n=16), clinical infections (n=20), clinical surveys (n=36), and community surveys (n=36) (Table 1). The genotypes of these isolates have been described previously (*11*,*12*). Strains were considered epidemic if they were isolated from patients treated in the same hospital, in the same ward and with an overlapping time-relationship, and if AFLP patterns showed at least 90% similarity (*12*). Epidemic isolates were recovered from clinical sites, blood and urine, as well as from feces. Only one representative isolate from each outbreak was used for analysis. The number of patients involved in each outbreak varied from 4 to >50 (*12*,*19*–*23*). Isolates were considered to be derived from a clinical infection if obtained from a clinical specimen, such as the blood, urine and wounds. All surveillance isolates, from patients and healthy persons, were isolated from fecal samples. Surveillance isolates were either from the community or from clinical surveillance when obtained from hospitalized patients. The hospital-stay duration of these patients, when cultures were obtained, was not available.

**Table 1:** Description of studied *Enterococcus faecium* isolates

Origin	Country	n
**Vancomycin-resistant *E. faecium***		
Epidemic	United Kingdom	1
	Netherlands	4
	United States	11
Clinical infections	Austria	1
	United Kingdom	7
	Israel	2
	Italy	1
	Netherlands	8
	United States	1
Clinical surveillance	Germany	1
	France	4
	United Kingdom	1
	Israel	1
	Italy	1
	Netherlands	27
	Slovenia	1
Community surveillance	United Kingdom	1
	Netherlands	35
**Vancomycin-susceptible *E. faecium***		
Clinical infections	Austria	6
	Belgium	1
	Switzerland	3
	Germany	14
	Spain	8
	France	8
	United Kingdom	1
	Greece	1
	Italy	16
	Poland	5
	Portugal	5
	Turkey	5
Clinical surveillance	Netherlands	5
Community surveillance	Netherlands	14

Isolates of VSEF (n=92) were derived from clinical infections (n=73), clinical surveys (n=5), and community surveys (n=14). The isolates from clinical infectious sites were obtained from the SENTRY Antimicrobial Surveillance Program, and originated from different hospitals in several European countries (Portugal, Germany, United Kingdom, France, Spain, Italy, Austria, Turkey, Switzerland, Greece, and Poland). Fifty-seven strains were blood isolates, 5 were isolated from urine, 8 from wounds, and 2 from respiratory tract specimens. Patient information was not available. All VSEF isolates derived from clinical surveys of fecal samples were from the University Hospital Maastricht. All VSEF isolates from community surveys of fecal samples were collected in the Netherlands. All bacterial isolates were collected during the 1990s.

### Identification and Susceptibility Testing

Enterococci were identified to the species level and were tested for the presence of the *vanA* gene by using a multiplex PCR described by Dutka-Malen et al. (*24*). Vancomycin and ampicillin/amoxicillin susceptibilities were determined by standard agar dilution methods, according to the National Committee for Clinical Laboratory Standards (NCCLS) guidelines (*25*). We considered MICs >16 μg/mL for ampicillin or amoxicillin and >8 for vancomycin to be resistant.

### Esp *PCR*

All strains were screened for *esp* by PCR, with two different primer sets (esp 11 [5′-TTGCTAATGCTAGTCCACGACC-3′] to esp 12 [5′-GCGTCAACACTTGCATTGCCGAA-3′] and 14F [5′-AGATTTCATCTTTGATTCTTGG-3′] to 12R [5′-AATTGATTCTTTAGCATCTGG-3′]). PCR conditions included an initial denaturation at 95°C for 15 min for activation of the HotStarTaq DNA polymerase (QIAGEN GmbH, Hilden, Germany), followed by 30 cycles of 94°C for 30 sec, 52°C for 30 sec, and 72°C for 1 min, followed by an extension at 72°C for 7 min. Reactions were performed in 25 μL by using the HotStarTaq Master Mix (QIAGEN GmbH). Strains negative in PCR were checked for the presence of the *esp* gene by Southern hybridization, as described previously (*12*). For this check, we generated an *esp*-specific probe (956 bp) using primers esp 11 and esp 12 (see above).

### Sequencing *PurK*

The *purK* gene encodes a phosphoribosylaminoimidazole carboxylase ATPase subunit involved in purine biosynthesis and is one of the seven housekeeping genes selected for multilocus sequence typing of *E. faecium* (*13*). A 492-bp fragment of the *purK* gene of a selection of strains, divided over all genogroups, was sequenced by using primers 5′-GCAGATTGGCACATTGAAAGT-3′ and 5′-TACATAAATCCCGCCTGTTTC/T-3′. PCR conditions included an initial denaturation at 95°C for 3 min, followed by 35 cycles of 94°C for 30 sec, 50°C for 30 sec, and 72°C for 30 sec, followed by an extension at 72°C for 5 min. Reactions were performed in 50 μL by using buffers and Taq polymerase (SphaeroQ, Leiden, Netherlands). The PCR products were purified with a PCR purification kit (QIAGEN GmbH) according to the manufacturer’s instructions. Subsequently, purified PCR products were sequenced directly with the ABI PRISM Big Dye Terminators cycle sequencing kit on an ABI PRISM DNA analyzer (Applied Biosystems, Foster City, CA). Sequences were aligned with BioNumerics (v. 2.5, Applied Maths, Kortrijk, Belgium) software.

### AFLP

AFLP typing and computer analysis of AFLP-generated patterns of VSEF was done as described previously (*11*) with minor modifications. Briefly, chromosomal DNA was digested with *Cfo*I and *Eco*RI and ligated to a single adapter with *Cfo*I and *Eco*RI protruding ends in a simultaneous reaction, followed by PCR using adapter-specific primers. The amplification products were separated and detected by using POP6-polymer on an ABI PRISM 3700 DNA Analyzer (Applied Biosystems). For each sample, 1 μL of the PCR reaction mixture (8 x diluted) was added to 9 μL of Hi-Di Formamid containing 12.5 μL/mL of the internal size marker (GeneScan-500–labeled with the red fluorescent dye 6-carboxy-x-rhodamine) in a MicroAmp Optical 96-well reaction plate (Applied Biosystems). The analyses were run in 3 hours. Genescan software (Applied Biosystems) was used for collection of data during the analysis and the data were subsequently exported into BioNumerics (Applied Maths) for further analysis. The Pearson product moment correlation coefficient was calculated, and the unweighted pair group method with arithmetic averages was used for cluster analysis. Using this methodology, we described four genogroups of VREF (*11*). We analyzed all isolates of VSEF and defined a cluster of isolates as a set of individual strains with AFLP patterns that shared at least 65% of the banding patterns (criterion defined for four genogroups [*11*]). Subsequently, to determine the matching genogroup, we compared AFLP banding patterns of each individual VSEF isolate with AFLP banding patterns of a library of 404 VREF, representing the four different AFLP genogroups. The library included VREF recovered from pigs (n=108) and nonhospitalized persons (n=28) as representatives of genogroup A, and strains from poultry (n=32), hospitalized patients (n=196), and calves (n=40), representing genogroups B, C, and D, respectively (*11*,*12*).

VSEF isolates were identified by using the identification module in BioNumerics. The genetic distance used for further analysis is 100 minus the calculated Pearson product moment correlation similarity coefficient. The degree of matching was expressed by an identification factor, which is the quotient of the average genetic distance between the tested strain and each of the isolates in the genogroup divided by the average genetic distance within a genogroup. If the average distance of the tested strain to each of the genogroup members is almost equal to the average distance among all strains in a genogroup, the identification factor will approach the value of one. So the lower the value of the identification factor, the more likely the test strain belongs to a particular genogroup.

## Results

**Figure 1:**
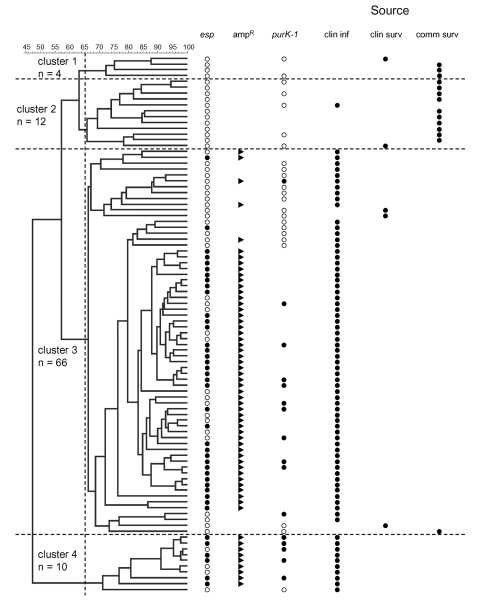
Cluster analysis of vancomycin-susceptible *Enterococcus faecium* (VSEF) isolates originating from clinical infections and clinical and community surveys. VSEF (n=92) were genotyped by amplified fragment length polymorphism (AFLP). Grouping of AFLP patterns showed four different clusters with >65% similarity. Numbers on the horizontal axis indicate percentage similarity. Closed circles indicate presence of the *esp* gene and the *purK*-1 allele and also the source of the isolates. Open circles represent *esp*-negative strains and other *purK* alleles. Closed arrowheads indicate ampicillin resistance. The vertical dashed line indicates the level of 65% similarity. Horizontal dashed lines indicate the boundaries of the four different clusters.

Using our predefined cutoff points for cluster analysis, we identified four clusters of VSEF (Figure 1). VSEF clustering seemed to be source-related. Clusters 1 (n=4) and 2 (n=12) contained surveillance isolates from community sources predominantly. All but one of the clinical infections isolates belonged to clusters 3 (n=66) and 4 (n=10), respectively.

On the basis of our calculations of the identification factors of VSEF and representative isolates of different VREF genogroups, we found that isolates of cluster 1 (n=4) resembled those of genogroup A, previously allocated to nonhospitalized patients and pig-derived VREF (Table 2). Similarly, isolates of cluster 2 (n=12) fitted best in genogroup B. Isolates from cluster 3 (n=66) showed almost equal resemblance to isolates from genogroups B and C, and isolates from cluster 4 (n=10) were also most identical to isolates from genogroup C (Table 2). The relationship between AFLP clusters of VSEF and sources was congruent with the previously described clustering of VREF. Almost all VSEF isolates (99%) derived from clinical infections clustered in clusters 3 and 4, clearly distinct from most VSEF isolates from community surveys (93%), which were found in clusters 1 and 2. A similar distribution was found previously among VREF with most (60%) isolates from clinical infections in genogroup C and most (89%) isolates from community surveys in genogroup A (*11*).

**Table 2:** Mean identification factor calculated for 92 vancomycin-susceptible *Enterococcus faecium* (VSEF) in clusters 1, 2, 3, and 4 to the vancomycin-resistant *E. faecium* (VREF) genogroups A–D

Cluster	n	Genogroup A	Genogroup B	Genogroup C	Genogroup D
		IF^a^	IF	IF	IF
1	4	**1.48±0.06**	1.76±0.16	2.5±0.16	3.93±0.31
2	12	2.25±0.20	**1.53±0.10**	2.12±0.15	3.33±0.16
3	66	2.98±0.10	**1.58±0.07**	**1.72±0.09**	3.97±0.06
4	10	3.91±0.45	2.36±0.29	**1.39±0.05**	4.74±0.32

^a^The mean identification factor (IF) represents a measure of how well the entries of the four different VSEF clusters belong to one of the previously described VREF genogroups, taking into consideration the internal spread of the VREF genogroups. Mean identification factor was determined by calculating the arithmetic averages of identification factors of isolates of a given cluster. The lowest identification factor (indicated in boldface type) represents the highest probability that isolates in a given VSEF cluster belong to a certain VREF genogroup. The 95% confidence limits are shown for each identification factor.

**Figure 2:**
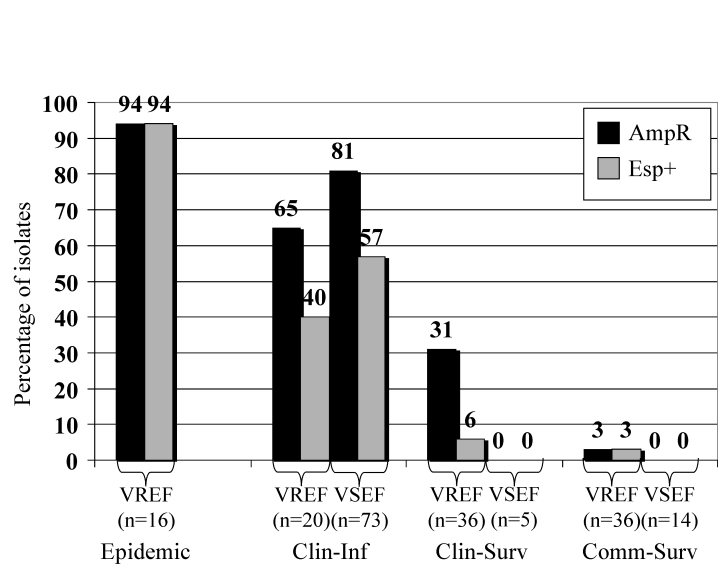
Frequencies of the *esp* gene and ampicillin resistance among vancomycin-susceptible enterococci (VSE) and vancomycin-resistant enterococci (VRE) of different origin. Percentages of *esp*-positive (solid bars) and ampicillin-resistant (dotted bars) VRE and VSE isolates originating from four different sources have been indicated. Clin-Inf, clinical infectious; Clin-Surv, clinical survey; Comm-Surv, community survey.

The presence of the variant *esp* gene in VREF and VSEF was strongly associated with a specific epidemiologic source because the presence of *esp* is higher in clinical infections and epidemic-associated isolates than in surveillance isolates (Figure 2). VREF isolates associated with nosocomial outbreaks were *esp*-positive, except for one. Prevalences of the variant *esp* gene in clinical infectious isolates were 57% and 40% for VSEF and VREF, respectively (p=ns). Prevalence of the variant *esp* gene in clinical and community survey isolates was low among VREF (6% and 3%, respectively) and completely absent among the 19 VSEF isolates tested.

Associations similar to the variant *esp* gene were found between ampicillin resistance and epidemiologic source for enterococcal isolates (Figure 2). All isolated associated with nosocomial VREF-outbreaks but one were resistant to ampicillin, as were 81% and 65% of infectious isolates of VSEF and VREF, respectively. Thirty-one percent of 36 nosocomial surveillance isolates of VREF were ampicillin resistant, as compared to none of five VSEF isolates obtained by clinical surveillance (p=ns). Finally, all but one isolate of VREF (n=36) and all VSEF isolates (n=14) obtained by surveillance of healthy persons were susceptible to ampicillin. When we combined these data, we found strong associations between the presence of the variant *esp*-gene and ampicillin resistance, both in VREF and VSEF: 98% of *esp*-positive VSEF and 92% of *esp*-positive VREF were resistant to ampicillin, as compared to 37% *esp*-negative VSEF and 20% *esp*-negative VREF isolates (p<0.0001).

The *purK* housekeeping gene was sequenced in 103 isolates: 64 VREF and 39 VSEF. The previously described type 1 allele was found in 39 isolates: 23 VREF and 16 VSEF. This specific allele type was associated with the presence of the variant *esp-*gene and ampicillin resistance but not with vancomycin resistance. The variant *esp*-gene was found in 25 (64%) of 39 isolates containing the *purK* type 1 allele and in only 1 (2%) of 64 isolates carrying other *purK* alleles (p<0.0001). Similarly, ampicillin resistance was detected in 36 (92%) of 39 isolates with *purK* type 1 allele and in only 5 (8%) of 64 isolates with other allele types (p<0.0001). In contrast, the *vanA* transposon was present in 23 (59%) of 39 isolates with *purK* type 1 allele and in 41 (64%) of 64 isolates with other allele types (p=ns).

## Discussion

Our study demonstrates the genetic relatedness of clusters of isolates of vancomycin-resistant and -susceptible *E. faecium* strains from different epidemiologic sources and provides evidence for selection of an *E. faecium* subtype associated with hospital outbreaks. This subtype is characterized by the presence of both ampicillin resistance and the variant *esp* gene. Furthermore, our findings suggest random horizontal spread of the *vanA* transposon to multiple genogroups of *E. faecium*. We hypothesize that the rise in infections caused by VREF resulted from nosocomial selection of a specific ampicillin-resistant *E. faecium* genotype harboring the variant *esp* gene and subsequent horizontal transfer of the *vanA* transposon.

Our study confirms that the previously demonstrated dichotomy between VREF isolated from healthy persons and patients (*11*) also exists for vancomcyin-susceptible *E. faecium* isolates. In VREF isolates, we could identify four genogroups, which were associated with particular hosts and environments and in which most isolates from healthy persons clustered distinctly from patient isolates. We showed that vancomycin-susceptible isolates clustered into three of these groups and that VSEF isolates from healthy persons also clustered distinctly from patient isolates. The genetic relationship between isolates and the genetic distinction between the four genogroups were based on AFLP analysis. We recently confirmed these findings with multilocus sequence typing (MLST) (*13*). Other researchers have also demonstrated host specificity of *E. faecium*. Quednau et al. suggested host specificity of isolates from chicken, pork, and humans by comparing restriction endonuclease profiles (*26*). In contrast, a recent study by Vancanneyt et al. also used AFLP; they did not confirm host-specific clustering of *E. faecium* (*27*). They described two main genomic groups in a population of 78 *E. faecium* strains isolated from seven European countries, and both groups comprised strains from (healthy) humans, animals, and food. All human clinical strains clustered in the largest genogroup, as did all strains (n=16) containing the *vanA* gene. Our findings that the *vanA* transposon was present in isolates of all genogroups proves that acquisition of this transposon was not influenced by and did not affect the preexisting relationship between the bacterium and host, which is a result of a long-term coevolution and mutual adaptation.

The presence of four genogroups in *E. faecium* seems to parallel the phylogenetic structure in *E. coli*, in which four ancestral groups (A, B1, B2, and D) have been described with some level of host specificity (*28*). However, in contrast to our finding of a single genetic lineage in *E. faecium* related to clinical symptoms and carrying the *esp* virulence gene, clinical isolates of *E. coli* are more widely distributed among the different ancestral groups (*29*). Furthermore, MLST of pathogenic *E. coli* strains showed that different ancestral lineages have acquired the same virulence factors (*30*), indicating that pathogenic potential in *E. coli* is not confined to a single ancestral lineage, which is suggested by our findings for *E. faecium*. Human and animal pathogenic *E. coli* strains share closely related genotypes and carry similar virulence factor profiles, suggesting that certain *E. coli* strains are pathogenic for both animals and humans (*31*). Whether this holds true for pathogenic *E. faecium* strains is unknown.

We recently found that the presence of the variant *esp*-gene is associated with nosocomial outbreaks of VREF in three continents, although this gene was not found in VREF strains isolated from healthy persons or animals (*12*). The outbreak strains were also characterized by a specific allele type of the *purK* gene, one of the housekeeping genes sequenced in the MLST method (*13*). Recently, other investigators reported the presence of the variant *esp* gene in clinical isolates of VSEF, demonstrating that this gene is not linked specifically to the *vanA* transposon (*15*–*18*). The findings of our study confirm the strong association between the presence of the *esp* gene and the relation with hospital outbreaks and clinical infections among patients with VREF as well as VSEF. Although the *esp* gene is virtually absent among community isolates, the presence of *esp* among VSEF and VREF from clinical infectious sites apparently unrelated to hospital outbreaks implies that this gene is not exclusively related to epidemic strains. Excluding the outbreak potential of the *esp*-positive VSEF strains in this and other studies is difficult. Only few outbreaks with VSEF have been documented (*32*–*34*). We have investigated and could not demonstrate the presence of the variant *esp* gene in isolates from one hospital outbreak of ampicillin-resistant *E. faecium* in Norway (data not shown).

Little is known about the function of the variant esp-gene, although epidemiologic findings support its role as a virulence factor. In *E. faecalis*, the homologue of this gene encodes for the enterococcal surface protein, and the presence of this gene has been associated with enhanced adherence capacities to uroepithelial surfaces, but not with increased virulence, in a mice model (*35*). Moreover, in *E. faecalis*, the *esp*-gene was highly associated with biofilm-formation capacity (*36*). Increased adherence capacities and biofilm formation of *esp*-positive *E. faecium* strains might explain its association with hospital outbreaks.

Like the variant *esp* gene, ampicillin resistance was found more frequently in isolates associated with infections and nosocomial outbreaks, both in VSEF and VREF. This source-relationship is probably caused by selective pressure of β-lactam antibiotics that are used extensively in hospitals. Emergence of ampicillin resistance in *E. faecium* was already demonstrated in the early 1980s and seemed to precede the emergence of vancomycin resistance by 10 years (*2*). A correlation between high prevalences of the *esp* gene and antibiotic resistance among *E. faecium* isolates from hospitalized patients was also reported recently by Coque et al. (*17*).

**Figure 3:**
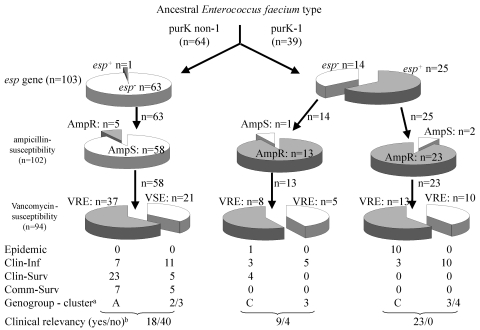
Hypothetical evolutionary scheme for *Enterococcus faecium* genotypes and phenotypes from an ancestral *E. faecium* type. Open slices indicate *esp*-negative, ampicillin-susceptible, and vancomycin-susceptible. Closed slices indicate *esp*-positive, ampicillin-resistant, and vancomycin-resistant. Numbers indicate the number of strains. Arrows indicate the putative evolutionary direction. Clin-Inf, clinical infectious; Clin-Surv, clinical survey; Comm-Surv, community survey. ^a^, dominant genogroup (A,C) for vancomycin-resistant enterococci and dominant cluster (2,3,4) for vancomycin-susceptible enterococci. ^b^, clinical relevant strains (“yes”) are the total of epidemic and clinical infectious isolates, clinical nonrelevant strains (“no”) are the total of clinical and community survey isolates.

Considering the sources of isolates, presence of ampicillin and vancomycin resistance, the presence of the variant *esp* gene, and the type 1 allele of the *purK* gene, we propose an evolutionary scheme for the specific genogroup of *E. faecium* associated with nosocomial outbreaks (Figure 3). However, our findings might have been biased by the composition of our collection of isolates and the fact that the *purK* was sequenced in a subset of all isolates. The *esp* gene and ampicillin resistance can obviously co-occur in a distinct genetic lineage of *E. faecium* characterized by the type 1 allele of the *purK* gene. We propose that *E. faecium* strains containing the type 1 allele of the *purK* gene have acquired the *esp* virulence gene and that this *E. faecium* genotype (*purK­*-1, *esp*-positive) is prominently present among clinical relevant strains and virtually absent among survey isolates. Yet another substantial part of the clinical relevant strains with genotype *purK*-1 do not carry the *esp* gene, which emphasizes that other virulence genes of *E. faecium* apart from *esp* are involved in the development of infections. Ampicillin resistance was predominantly found among the *purK*-1 genotype and is almost absent among other *E. faecium* genotypes. This occurrence of resistance is, presumably, the result of selective antibiotic pressure. Chromosomal linkage of the *purK*-1 allele, the variant *esp* gene, and ampicillin resistance could have promoted this selection. Finally, glycopeptide usage in and outside hospitals, both in humans and animals, resulted in the selection of vancomycin-resistant strains in both the *purK*-1 genotype and the other genotypes. The presence of similar proportions of vancomycin resistance in all genotypes probably reflects horizontal transfer of the vancomycin-resistance transposon. This hypothesis implies the development of a hospital-adapted genogroup of *E. faecium*, characterized by the type-1 allele of *purK*, the variant *esp*-gene and ampicillin resistance, which has spread unnoticed, thereby creating a pool of strains with epidemic potential. Only after becoming vancomycin-resistant has this genogroup become recognized as clinically relevant.
